# Exposure to paternal tobacco smoking increased child hospitalization for lower respiratory infections but not for other diseases in Vietnam

**DOI:** 10.1038/srep45481

**Published:** 2017-03-31

**Authors:** Reiko Miyahara, Kensuke Takahashi, Nguyen Thi Hien Anh, Vu Dinh Thiem, Motoi Suzuki, Hiroshi Yoshino, Le Huu Tho, Hiroyuki Moriuchi, Sharon E. Cox, Lay Myint Yoshida, Dang Duc Anh, Koya Ariyoshi, Michio Yasunami

**Affiliations:** 1 Department of Clinical Tropical Medicine, Institute of Tropical Medicine, Graduate School of Biomedical Sciences, Nagasaki University, Nagasaki, Japan; 2 National Institute of Hygiene and Epidemiology, Hanoi, Vietnam; 3 Department of Labor, Invalids and Social Affairs of Khanh Hoa Province, Nha Trang, Vietnam; 4 Department of Pediatrics, Graduate School of Biomedical Sciences, Nagasaki University, Nagasaki, Japan; 5 Department of Global Health, School of Tropical Medicine and Global Health, Nagasaki University, Nagasaki, Japan; 6 Department of Population Health, London School of Hygiene and Tropical Medicine, London, UK; 7 Department of Pediatric Infectious Diseases, Institute of Tropical Medicine, Nagasaki University, Nagasaki, Japan

## Abstract

Exposure to environmental tobacco smoke (ETS) is an important modifiable risk factor for child hospitalization, although its contribution is not well documented in countries where ETS due to maternal tobacco smoking is negligible. We conducted a birth cohort study of 1999 neonates between May 2009 and May 2010 in Nha Trang, Vietnam, to evaluate paternal tobacco smoking as a risk factor for infectious and non-infectious diseases. Hospitalizations during a 24-month observation period were identified using hospital records. The effect of paternal exposure during pregnancy and infancy on infectious disease incidence was evaluated using Poisson regression models. In total, 35.6% of 1624 children who attended follow-up visits required at least one hospitalization by 2 years of age, and the most common reason for hospitalization was lower respiratory tract infection (LRTI). Paternal tobacco smoking independently increased the risk of LRTI 1.76-fold (95% CI: 1.24–2.51) after adjusting for possible confounders but was not associated with any other cause of hospitalization. The population attributable fraction indicated that effective interventions to prevent paternal smoking in the presence of children would reduce LRTI-related hospitalizations by 14.8% in this epidemiological setting.

Infectious diseases such as pneumonia and diarrhoea are major contributors to child morbidity and mortality in low- and middle-income countries, with most cases occurring in the first 2 years of life^1^. These diseases are associated with a high economic burden^2^,^3^,^4^. In Southeast Asia, after excluding neonatal deaths, pneumonia and diarrhoea account for half of all deaths in children under 5 years of age^5^.

Environmental tobacco smoke (ETS) exposure is an important and early modifiable risk factor for childhood illnesses such as respiratory symptoms^6^,^7^,^8^,^9^,^10^, respiratory infections^11^,^12^,^13^, asthma^14^,^15^,^16^ and sudden infant death^17^. The biological mechanisms of the disease risk conferred by ETS exposure have been investigated by *in vitro* functional studies on Toll-like receptors^18^,^19^ and blood cells^20^, and these studies have suggested that tobacco smoke alters local and systemic innate immunity. Therefore, the effect of ETS exposure may not be limited to respiratory diseases. However, there is limited and conflicting evidence regarding the association of ETS exposure with the risks of other types of infectious diseases such as acute otitis media^21^ and gastroenteritis^22^,^23^,^24^,^25^. Furthermore, few prospective studies of child populations have assessed the risks associated with ETS exposure in populations in which the contribution of female or maternal smoking exposure is negligible like Vietnamese^26^. We previously reported that ETS exposure due to household members was significantly associated with an increased risk of parent-reported child pneumonia (OR = 1.55, 95% CI: 1.25–1.92) in a cross-sectional population surveillance study in Vietnam^27^; however, the detection of cases was not fully reliable because it was based on parental self-report questionnaires. As tobacco smoking is highly prevalent among young Vietnamese men and as only 1.0% of smokers in Nha Trang, Vietnam, are women 18–50 years of age^27^, parental smoking, particularly paternal smoking, could be a risk factor for childhood diseases including, but not limited to, respiratory infections.

In the present study, we described the burden and pattern of severe illness including infectious and non-infectious diseases that require hospitalization using the medical records of Vietnamese children registered in a prospective birth cohort study. Additionally, we aimed to improve the strength of evidence for the association of parental smoking with the incidence of childhood health problems.

## Results

### Baseline characteristics of the Nha Trang birth cohort

We enrolled 1999 children at birth; 1624 of those children (81.2%) were confirmed to have been residing in the study catchment area for 24 months or from birth to death. The remaining 375 children were excluded from the analysis: 5 died, with uncertain information about the date of death, the cause of death or both; 251 had left the study catchment area; and 119 could not be traced. Children who attended follow-up visits had a significantly higher household economic status, higher mother’s education level, older maternal age and a higher likelihood of living with siblings than children who did not (Supplementary Table S1). The prevalence of exposure to paternal tobacco smoking during pregnancy and infancy (PS_p/i_) was 57.3%. None of the women reported smoking tobacco during pregnancy (Table 1), and ETS exposure was not caused by maternal smoking in this population. Exposure to PS_p/i_ was associated with young maternal age, low household income, low maternal education level, living with siblings and low weight gain during pregnancy (Table 1).

### Incidence of hospitalizations

A total of 939 hospitalization events were recorded during the 24-month observation period. The most common cause of these hospitalizations was lower respiratory tract infections (LRTIs) (11.1% of the study participants), accounting for 24.7% of all hospitalizations. Gastrointestinal infections (GIs) and non-focal viral infections were the second and third most common causes of hospitalization, accounting for 20.8% and 17.4% of all hospitalizations, respectively (Table 2). The median length of hospital stay for LRTIs was 4 days, which was longer than that of other infectious diseases (3 days, p < 0.001 by Kruskal-Wallis test) including GIs (LRTIs vs GIs, Wilcoxon rank sum test p < 0.001) and upper respiratory tract infections (URTIs) (LRTIs vs URTIs, Wilcoxon rank sum test p < 0.001).

### Risk factor analysis of cause-specific hospitalizations

**Figure 1:**
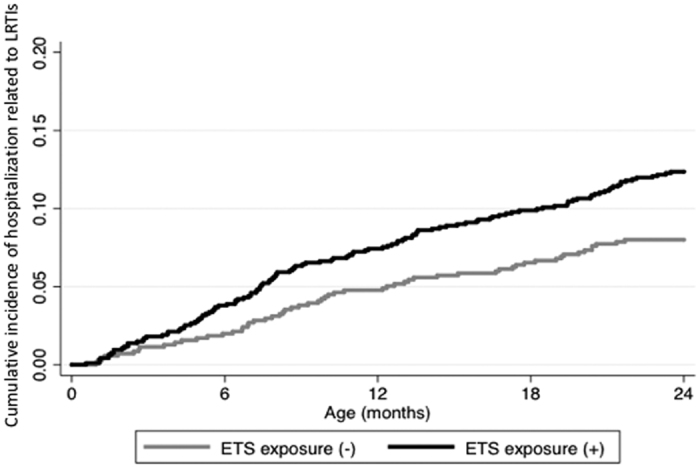
Kaplan-Meier plot of cumulative incidence of hospital admissions due to lower respiratory tract infections by environmental tobacco exposure. Cumulative incidence (events per person) of hospitalizations related to lower respiratory tract infections (LRTIs) during the observation period was plotted for the children who were exposed to environmental tobacco smoking, ETS exposure (+) and those who were not, ETS exposure (−).

The results of univariable analyses for cause-specific hospitalization are shown in Supplementary Tables S2 and S3. PS_p/i_ was identified as a risk factor for LRTIs but not for other causes of hospitalization, whereas age at hospitalization, calendar month at hospitalization, living with a sibling, maternal age at delivery and maternal body mass index were associated with other types of infectious and non-infectious diseases but not with LRTIs. In the multivariable analyses, exposure to PS_p/i_ independently increased the risk of total infectious disease-related hospitalizations 1.26-fold (p = 0.012); PS_p/i_ increased the risk of LRTIs 1.76-fold (95% C.I. 1.24–2.51; p = 0.002), whereas the incidence of other infectious diseases including URTIs as well as non-infectious diseases was not associated with PS_p/i_ (Table 3). LRTIs represented the increase in infectious disease risk due to PS_p/i_. Although maternal age at delivery, low birth weight (LBW, less than 2.5 kg) and monthly household income were possible risk factors for LRTIs in the univariable analysis (p < 0.1), the effect size of PS_p/i_ on LRTIs did not change after adjusting for these potential confounders, confirming that the effect of PS_p/i_ was independent of LBW and other residual confounders. The population attributable fraction (PAF) of all infections associated with PS_p/i_ was 11.8%, and the PAF of LRTIs associated with PS_p/i_ was 14.7%. PS_p/i_ exposure increased the population attributable risks (PARs) of all infections and of LRTIs by 48.9 per 1000 person-years of observation (PYO) and 39.4 per 1000 PYO, respectively. Although LBW was a strong risk factor for LRTI-related hospitalizations, increasing the risk 2.27-fold (p = 0.062), LBW increased the PAF of LRTIs by 1.3%. Thus, LBW had a smaller impact on LRTI incidence than PS_p/i_. A Kaplan-Meier plot depicted the hospitalization events related to LRTI that occurred in the early months of PS_p/i_-exposed children’s lives, and the effect of PS_p/i_ persisted during the entire observation period (Fig. 1).

## Discussion

In the present study, 35% of the children were hospitalized at least once during their first 2 years of life, and 80% of the hospitalizations were caused by common infectious diseases such as LRTIs, GIs and non-focal viral infections. ETS exposure due to PS_p/i_ was associated with significantly increased risks of infectious disease-related hospitalization and, more specifically, LRTI-related hospitalization. However, the influence of PS_p/i_ on the risk of other infectious and non-infectious diseases was not demonstrated in the same population.

We found that PS_p/i_ independently increased the risk of LRTI-related hospitalization in this prospective cohort study after controlling for low socio-economic status, young maternal age, low weight gain during pregnancy and LBW, although these confounders might increase the rate of hospitalization. This finding is consistent with the results of our previous cross-sectional study, which showed that exposure to in-house tobacco smoking in the same province increased the prevalence of pneumonia in children under 5 years of age (adjusted OR = 1.55, 95% CI: 1.25–1.92)^27^. These results are also consistent with the findings of a study performed in Hong Kong that assessed the association of ETS exposure with respiratory tract or febrile illness (OR 1.28, 95% CI: 1.01–1.61); most of the smoking exposure in that setting was also due to fathers and other household members^13^.

Maternal smoking exposure *in utero* and during postnatal periods has been shown to present a risk of childhood diseases; maternal smoking during pregnancy has been associated with a decrease in lung function^28^ and an increased incidence of wheezing and asthma^29^. Although PS_p/i_ was not found to influence respiratory symptoms^7^, acute respiratory infections^12^ or respiratory function^30^ among young children in some studies, the importance of PS_p/i_ has been shown in other studies. A large Norwegian cohort study suggested that paternal smoking was significantly associated with a risk of LRTIs among children 6–18 months of age after adjusting for maternal smoking^6^, although the effects were smaller than those of maternal smoking during pregnancy. In the present study, we were able to directly evaluate the effect of PS_p/i_ without adjusting for the effect of maternal smoking exposure.

In the Norwegian study mentioned above, both prenatal maternal smoking exposure and postnatal paternal smoking exposure without maternal smoking increased the risk of LRTIs, but prenatal paternal exposure *in utero* did not^6^. Fuentes-Leonarte *et al*. reported that postnatal exposure from fathers was related to otitis^31^. In our study, paternal smoking status was determined at enrolment and we did not distinguish the effects of ETS exposure during pregnancy from those in infancy, because we assumed that smoking habits would not change after a child’s birth or during childhood. Given the similar rates of smoking status between 2006 and 2010 found in census data (Supplementary Table S4), misclassification of paternal smoking exposure after birth would not be frequent. The effect of second-hand smoking during pregnancy and infancy could be clarified by measuring the urinary cotinine levels of mothers and children, as these measurements would provide more objective data on ETS exposure status^32^,^33^,^34^,^35^.

Previous studies have shown that maternal smoking during pregnancy is a risk factor for LBW and that paternal smoking has a smaller effect on LBW^36^,^37^,^38^. In the present study, we also found that there were more LBW children among those exposed to paternal smoking than in those without exposure (2.6% vs. 1.9%), although this difference did not reach significance (p = 0.345).

The high burden of hospital admissions due to respiratory infections (LRTIs + URTIs, 110.9 per 1000 PYO) among children under 2 years of age in Vietnam was similar to the burden of acute respiratory infections identified among children under 1 year old in a previous study conducted in both an urban area, Ho Chi Minh City (81 per 1000 PYO), and a rural area, Dong Thap (138 per 1000 PYO)^39^. Preventive measures against second-hand smoking during pregnancy and infancy are expected to have a high impact nationwide, not only in Nha Trang, because of the high incidence rate of respiratory infections throughout Vietnam.

Our study has several limitations. First, we captured only hospitalized cases so that the results might be hampered by the selection bias: factors influencing on their medical care-seeking behaviours might be associated with the number of patients with serious illness who did not consult a doctor at the hospital. It would not be true because children can receive free medical treatments at public hospitals like KHGH. This notion was supported by our previous cross sectional surveillance in the province. Second, the impact of paternal smoking exposure might be underestimated in the present study because the children who were lost to follow-up and the PS_p/i_-exposed children shared similar characteristics, such as low socio-economic status, young maternal age and living with siblings. However, the percentage of children exposed to PS_p/i_ did not differ between those followed and those lost to follow-up (60.8% vs. 57.3%, p = 0.211, Supplementary Table S1), mitigating the risk of this limitation. Another possible source of underestimation of impact of paternal smoking comes from an absence of information on the exposure to tobacco smoking of other members of household without PS_p/i_. However, as we previously reported, parental smoking accounted for a large portion (73.5%) of tobacco smoke in households among the children aged <5 years lived in the province in 2006^27^. Third, there are additional potential confounding factors that we did not address in this study such as crowding and sanitation^40^,^41^. The major strength of this study was its prospective study design and relatively high follow-up rate, which minimized the selection and recall biases inherent to retrospective studies. As Khanh Hoa General Hospital (KHGH) is the only tertiary health facility available to the study population, and as the study population has good access to primary and secondary health facilities for referral, we were able to capture almost all severe illness events.

PS_p/i_ contributed to the incidence of severe LRTIs requiring hospitalization in children less than 2 years old in Nha Trang, Vietnam, and was responsible for 24% of the total LRTI incidence; in contrast, LBW was responsible for only 1.3% of all LRTI cases. As LRTIs are the most common cause of hospitalization and are associated with longer hospital stays, smoking reduction interventions targeted at households with pregnant women and children may effectively improve child health.

## Methods

### Study area and data collection

Nha Trang, the capital of Khanh Hoa province, central Vietnam, is the seventh most populated city in the nation (URL: http://www.citypopulation.de/Vietnam.html). Prior to the present study, we conducted a cross-sectional surveillance of 74,228 households in 33 urban and rural communities (communes) in this province to collect information about the demographics, socioeconomic status and smoking habits of household members; the data were collected from the heads of households by trained interviewers during June and July of 2006. Census data were updated by interview in 2010.

We initiated the Nha Trang Birth Cohort study in 16 urban and semi-urban communes in Nha Trang in May 2009. The catchment area was determined by excluding communes in business districts of the city centre and rural communes located far from the city centre to maintain uniform health care-seeking behaviour. All pregnant women over 18 years of age living in the study communes who were admitted on weekdays to the obstetric ward of KHGH, the only hospital providing in-patient paediatric care in this area, were invited at admission to participate in a prospective cohort study between May 2009 and May 2010. Pregnant women who had serious complications before or during pregnancy, who had multiple gestations, who gave birth at gestational age <22 weeks and who had a stillbirth were excluded from this study. Prior to delivery, we obtained informed consent from the eligible mothers and collected maternal blood samples to measure haemoglobin levels. Delivery and birth information was collected from hospital records. Trained medical staff interviewed the mothers to obtain baseline parental health, socio-economic status and demographic information using fixed questionnaires within 2 days of delivery, and the staff also asked about parental tobacco smoking history and smoking status during pregnancy to determine ETS exposure. We used smoking status at enrolment because it was assumed to be the best predictor of exposure to ETS during the 2-year follow-up period, based on the fact that over 60% of males aged 20–44 years maintained the same smoking habits between 2006 and 2010 (Supplementary Table S1). Two years after delivery, we contacted the parents and confirmed their full residence over the previous 24 months. The study was conducted in accordance with the World Medical Association’s Declaration of Helsinki (1975, as revised in 2008), and the study protocol was approved by the Institutional Review Board of Nagasaki University and the National Institute of Hygiene and Epidemiology of Vietnam (Reference no. 09031836).

### Event detection and case definition

All hospitalization events among the study participants from May 2009 to May 2012 were identified using KHGH patient records; these records also provided the information on the causes of hospital admission ascertained by paediatricians in the hospital based on International Classification of Diseases (ICD-10) codes (http://apps.who.int/classifications/icd/en/). In the present study, diagnoses were dichotomized into infectious and non-infectious diseases. Infectious diagnoses were further categorized into five groups: LRTIs, GIs, URTIs, non-focal viral infections and miscellaneous infections. Non-infectious diseases were classified into digestive system diseases, asthma, injuries and other diseases. Re-hospitalization for the same diagnosis as in a previous hospital admission was counted as an independent event if it occurred at least 14 days after the prior hospital admission.

Because we used medical record information to detect cases, we were unable to consistently confirm a definitive diagnosis of pneumonia on chest X-ray to determine the cause of hospitalization, as performed in other studies^42^,^43^. However, we conducted a pre-study assessment of the validity of respiratory tract infection diagnoses by asking an independent paediatrician from Japan to re-read the chest X-rays and to evaluate the hospital records; based on the available information, the independent paediatrician agreed with the diagnoses in nearly all cases.

### Statistical analysis

A descriptive analysis of the baseline socio-demographic factors was conducted for all enrolled children and for the children who attended follow-up visits in the Nha Trang Birth Cohort study. Children who attended follow-up visits and were confirmed to have lived within the study area for 24 months after birth were included in further analyses. We also compared the baseline characteristics of the parental smoking-exposed group with the non-exposed group as a main exposure variable using Chi-square tests for categorical variables and t-tests for continuous variables. The incidence rates for infectious diseases (LRTIs, GIs, URTIs, non-focal viral infections and miscellaneous infections) and non-infectious diseases were calculated as the number of events divided by the number of PYO, adjusting for clustering by multiple episodes in an individual. The rate ratio was calculated to assess the effect of ETS exposure on hospitalization due to specific diagnosed diseases using a Poisson regression model. Age at hospital admission, sex and calendar month of hospital admission were included as confounders in the multivariate models. Further variables were included if their p-values were less than 0.1 in the univariate models, which used a Poisson regression model for the association between each diagnosed disease and baseline characteristics. Confidence intervals and p-values were calculated using cluster-robust standard errors to adjust for clustering by individuals with multiple episodes. The population attributable risks (PARs) were calculated by subtracting the incidence rate in the non-exposed population from the incidence rate in the exposed population (per 1000 PYO). The population attributable fraction (PAF) was calculated using the following formula: PAF = p (θ − 1)/θ, where p = the proportion of patients who were exposed and θ = adjusted relative risk. The median length of hospital stay was compared between patients with different diagnoses using the Kruskal-Wallis test and between any two groups using the Wilcoxon rank sum test. A time-to-event analysis was conducted to determine the existence of an age-dependent effect of a risk factor using Kaplan-Meier plots. Statistical tests were performed using STATA 12 (StataCorp LP, College Station, Texas, USA).

## Additional Information

**How to cite this article**: Miyahara, R. *et al*. Exposure to paternal tobacco smoking increased child hospitalization for lower respiratory infections but not for other diseases in Vietnam. *Sci. Rep.* **7**, 45481; doi: 10.1038/srep45481 (2017).

**Publisher's note:** Springer Nature remains neutral with regard to jurisdictional claims in published maps and institutional affiliations.

## Supplementary Material

Supplementary Information

## Figures and Tables

**Table 1:** Baseline characteristics of followed children in the Nha Trang Birth Cohort.

Variables	Category/unit	Total (N = 1624)	Paternal smoking (−) (N = 694)	Paternal smoking (+) (N = 930)	p-value^1^
Paternal smoking	Yes	930 (57.3%)	—	—	—
Maternal smoking	Yes	0 (0%)	0 (0%)	0 (0%)	—
Sex	Girls	786 (48.4%)	341 (49.1%)	445 (47.8%)	0.608
Birth weight	<2.5 kg	37 (2.3%)	13 (1.9%)	24 (2.6%)	0.345
Maternal age at delivery	<25 years	396 (24.4%)	152 (21.9%)	244 (26.2%)	
	25–34 years	994 (61.2%)	459 (66.1%)	535 (57.5%)	0.002
	≥35 years	234 (14.4%)	83 (12.0%)	151 (16.2%)	
Monthly household income^2^	x100,000 VND	51.6 (35.9)	53.9 (39.2)	49.9 (32.1)	0.030
Mother’s education level	More than 6 years	1,316 (81.0%)	605 (87.2%)	711 (76.5%)	<0.001
Siblings	Yes	841 (51.8%)	331 (47.7%)	510 (54.8%)	0.004
Weight gain during pregnancy	<10 kg	439 (27.0%)	150 (21.6%)	289 (31.1%)	
	10–15 kg	758 (46.7%)	349 (50.3%)	409 (44.0%)	<0.001
	≥15 kg	427 (26.3%)	195 (28.1%)	232 (25.0%)	
Mode of delivery^3^	NVD	598 (36.8%)	245 (35.3%)	353 (38.0%)	
	CS	699 (43.0%)	306 (44.1%)	393 (42.3%)	0.548
	Induction	327 (20.1%)	143 (20.6%)	184 (19.8%)	
Maternal anaemia (Hb level<11 mg/dl)^4^	Yes	416 (25.6%)	165 (23.8%)	251 (27.0%)	0.142
Maternal BMI^5^	<18.5 kg/m^2^	418 (25.7%)	175 (25.2%)	243 (26.1%)	0.704
	18.5–23.5 kg/m^2^	1,161 (71.5%)	502 (72.3%)	659 (70.9%)	
	≥23.5 kg/m^2^	45 (2.8%)	17 (2.5%)	28 (3.0%)	

^1^p-values were calculated using Chi-square tests between paternal smoking exposure (+) and paternal smoking exposure (−) except for monthly household income, for which two groups were compared using t-tests.

^2^Standard deviations are in parentheses; data were available for 1467 of 1624 children. VND: Vietnamese dong.

^3^NVD: normal vaginal delivery, CS: caesarean section.

^4^Hb: haemoglobin.

^5^BMI: body mass index.

**Table 2:** Hospital admissions observed in the Nha Trang Birth Cohort.

Diagnosis classification	ICD-10 code	Number of children	Number of events	Incidence rate (95% CI), per 1000 PYO	Length of hospital stay, days
					Median (min-max)
**Overall hospitalization**		**578**	**939 (100.0%)**	**296.4 (273.0**–**321.9)**	**3 (1–36)**
**Infections**		**495**	**745 (79.3%)**	**234.1 (214.4**–**255.7)**	**3 (1–31)**
Lower respiratory tract infections	J18, J20, J21	180	232 (24.7%)	71.6 (62.8–81.5)	4 (1–16)
Gastrointestinal infections	A03, A04, A05, A08, A09	179	196 (20.8%)	60.6 (52.7–69.8)	3 (1–31)
Non-focal viral infections	B09, B34, R50	144	163 (17.4%)	50.6 (42.9–59.6)	3 (1–14)
Upper respiratory tract infections	J02, J03, J04, J06	117	127 (13.5%)	39.3 (32.8–47.2)	3 (1–10)
Miscellaneous infections	A37, A38, A75, A87, A90, A91, B01, B05, H65, H66	22	27 (2.9%)	8.3 (5.7–12.1)	4 (1–8)
**Non-infectious diseases**		**163**	**194 (20.7%)**	**60.5** (**51.6**–**71.0)**	**3 (1**–**36)**
Diseases of the digestive system	K30, K59, K60, K40, K56, K13, K63, K92, K29, K61, R10, R11	59	65 (6.9%)	20.2 (15.5–26.3)	1 (1–19)
Injuries	S09, T31, W33, S01, S06, S51, S62, X64	19	19 (2.0%)	5.9 (3.7–9.2)	2 (1–15)
Asthma	J45	15	19 (2.0%)	5.9 (3.7–9.2)	4 (1–9)
All other diseases	Others^1^	83	91 (9.7%)	28.1 (22.6–34.9)	3 (1–36)

^1^The other diseases identified in the cohort were neonatal disorders (P07, P08, P36, P38, P59), anaemia (D50, D63, D64), other blood disorders (D66, D69, D69.8, D69.3, D75, D89, R79), skin diseases (L02, L02.4, L08, L50.0, M76.0), benign neoplasms (D18, D23, D36), urinary/kidney diseases (N28, N39), adenoids/chronic rhinitis (J31.1, J35.2), congenital malformations (Q21, Q35, Q54, Q79.2) and others (R53, R56.0, X23, G40, I84, H72.9).

**Table 3:** Unadjusted and adjusted rate ratios for the associations between childhood diseases and paternal smoking exposure during pregnancy and infancy in the Nha Trang Birth Cohort.

Outcome	Exposure to paternal smoking vs. Non-exposure to paternal smoking^1^			
	Unadjusted RR^2^	p-value	Adjusted RR	p-value
All infections	**1.22 (1.02–1.46)**	**0.032**	**1.26 (1.05–1.51)**^3^	**0.012**
Lower respiratory tract infections	**1.81 (1.30–2.52)**	**<0.001**	**1.76 (1.24–2.51)**^4^	**0.002**
Gastrointestinal infections	1.06 (0.80–1.41)	0.681	1.05 (0.79–1.39)^5^	0.748
Non-focal viral infections	0.83 (0.60–1.16)	0.283	0.87 (0.63–1.22)^6^	0.424
Upper respiratory tract infections	1.15 (0.79–1.67)	0.458	1.18 (0.81–1.72)^7^	0.377
Non-infectious diseases	1.25 (0.90–1.74)	0.198	1.24 (0.89–1.72)^8^	0.206

Statistically significant results are shown in bold. 95% confidence intervals of point estimates are in parentheses.

^1^Paternal smoking during pregnancy and infancy.

^2^RR: rate ratio.

^3^Rate ratio adjusted for calendar month, age at hospital admission, sex, low birth weight, maternal age at delivery and maternal BMI.

^4^Rate ratio adjusted for calendar month, age at hospital admission, sex, low birth weight, maternal age at delivery and monthly household income.

^5^Rate ratio adjusted for calendar month, age at hospital admission, and sex.

^6^Rate ratio adjusted for calendar month, age at hospital admission, sex, maternal BMI and sibling.

^7^Rate ratio adjusted for calendar month, age at hospital admission, sex and sibling.

^8^Rate ratio adjusted for calendar month, age at hospital admission, sex, sibling and maternal age at delivery.
