# Author Correction: Exposure to paternal tobacco smoking increased child hospitalization for lower respiratory infections but not for other diseases in Vietnam

**DOI:** 10.1038/s41598-020-62383-3

**Published:** 2020-04-06

**Authors:** Reiko Miyahara, Kensuke Takahashi, Nguyen Thi Hien Anh, Vu Dinh Thiem, Motoi Suzuki, Hiroshi Yoshino, Le Huu Tho, Hiroyuki Moriuchi, Sharon E. Cox, Lay Myint Yoshida, Dang Duc Anh, Koya Ariyoshi, Michio Yasunami

**Affiliations:** 1 Department of Clinical Tropical Medicine, Institute of Tropical Medicine, Graduate School of Biomedical Sciences, Nagasaki University, Nagasaki, Japan; 2 National Institute of Hygiene and Epidemiology, Hanoi, Vietnam; 3 Department of Labor, Invalids and Social Affairs of Khanh Hoa Province, Nha Trang, Vietnam; 4 Department of Pediatrics, Graduate School of Biomedical Sciences, Nagasaki University, Nagasaki, Japan; 5 Department of Global Health, School of Tropical Medicine and Global Health, Nagasaki University, Nagasaki, Japan; 6 Department of Population Health, London School of Hygiene and Tropical Medicine, London, UK; 7 Department of Pediatric Infectious Diseases, Institute of Tropical Medicine, Nagasaki University, Nagasaki, Japan

Correction to: *Scientific Reports* 10.1038/srep45481, published online 31 March 2017

This Article contains errors. The definition of population attributable risks (PARs) in the Methods, under the subheading ‘Statistical analysis’ is incorrect.

“The population attributable risks (PARs) were calculated by subtracting the incidence rate in the non-exposed population from the incidence rate in the exposed population (per 1000 PYO).”

should read:

“The population attributable risks (PARs) were calculated by subtracting the incidence rate in the non-exposed population from the incidence rate in the whole population (per 1000 PYO).”

As a result, in the Results section, under the subheading ‘Risk factor analysis of cause-specific hospitalizations’,

“The population attributable fraction (PAF) of all infections associated with PS_p/i_ was 11.8%, and the PAF of LRTIs associated with PS_p/i_ was 14.7%. PS_p/i_ exposure increased the population attributable risks (PARs) of all infections and of LRTIs by 48.9 per 1000 person-years of observation (PYO) and 39.4 per 1000 PYO, respectively.”

should read:

“The population attributable fraction (PAF) of all infections associated with PS_p/i_ was 11.8%, and the PAF of LRTIs associated with PS_p/i_ was 14.7%. PS_p/i_ exposure increased the population attributable risks (PARs) of all infections and of LRTIs by 26.0 per 1000 person-years of observation (PYO) and 22.5 per 1000 PYO, respectively.”

In addition, there is a typographical error in the Abstract,

“The population attributable fraction indicated that effective interventions to prevent paternal smoking in the presence of children would reduce LRTI-related hospitalizations by 14.8% in this epidemiological setting.”

should read:

“The population attributable fraction indicated that effective interventions to prevent paternal smoking in the presence of children would reduce LRTI-related hospitalizations by 14.7% in this epidemiological setting.”

Finally, there is a typographical error in the Discussion,

“PS_p/i_ contributed to the incidence of severe LRTIs requiring hospitalization in children less than 2 years old in Nha Trang, Vietnam, and was responsible for 24% of the total LRTI incidence; in contrast, LBW was responsible for only 1.3% of all LRTI cases. As LRTIs are the most common cause of hospitalization and are associated with longer hospital stays, smoking reduction interventions targeted at households with pregnant women and children may effectively improve child health.”

should read:

“PS_p/i_ contributed to the incidence of severe LRTIs requiring hospitalization in children less than 2 years old in Nha Trang, Vietnam, and was responsible for 14.7% of the total LRTI incidence; in contrast, LBW was responsible for only 1.3% of all LRTI cases. As LRTIs are the most common cause of hospitalization and are associated with longer hospital stays, smoking reduction interventions targeted at households with pregnant women and children may effectively improve child health.”

