# Supplementary material for: Exposure to paternal tobacco smoking increased child hospitalization for lower respiratory infections but not for other diseases in Vietnam
Source: Sci Rep. 2017 Mar 31;7:45481. doi: 10.1038/srep45481 (PMC5374438; doi:10.1038/srep45481)
Supplement: Supplementary Information [file srep45481-s1.pdf]

SUPPLEMENTARY INFORMATION

Title:

Exposure to paternal tobacco smoking increased child hospitalization for lower respiratory infections but not for other diseases in Vietnam

Authors:

Reiko Miyahara[1], Kensuke Takahashi[1], Nguyen Thi Hien Anh[2], Vu Dinh Thiem[2], Motoi Suzuki[1], Hiroshi Yoshino[1], Le Huu Tho[3], Hiroyuki Moriuchi[4], Sharon E Cox[5,6], Lay Myint Yoshida[7], Dang Duc Anh[2], Koya Ariyoshi[1] and Michio Yasunami[1,7]*

[1]Department of Clinical Tropical Medicine, Institute of Tropical Medicine, Graduate School of Biomedical Sciences, Nagasaki University, Nagasaki, Japan

[2]National Institute of Hygiene and Epidemiology, Hanoi, Vietnam

[3]Department of Labor, Invalids and Social Affairs of Khanh Hoa Province, Nha Trang, Vietnam

[4]Department of Pediatrics, Graduate School of Biomedical Sciences, Nagasaki University, Nagasaki, Japan

[5]Department of Global Health, School of Tropical Medicine and Global Health, Nagasaki University, Nagasaki, Japan

[6]Department of Population Health, London School of Hygiene and Tropical Medicine, London, UK

[7]Department of Pediatric Infectious Diseases, Institute of Tropical Medicine, Nagasaki University, Nagasaki, Japan

*Corresponding author:

E-mail: yasunami@nagasaki-u.ac.jp

His current affiliation is:

Life Science Institute, Saga-Ken Medical Centre Koseikan, Saga, Japan

Supplementary Table S1: Baseline characteristics of the children enrolled to the study.

Variables	Category/ unit	All enrolled (N=1,999)	Followed-up (N=1,624)	Lost to follow-up (N=375)	p-value[1]
Sex	Girls	966 (48.3%)	786 (48.4%)	180 (48.0%)	0.889
Birth weight	<2.5 kg	47 (2.4%)	37 (2.3%)	10 (2.7%)	0.655
Maternal age at delivery	<25 years	512 (25.6%)	396 (24.4%)	116 (30.9%)	0.026
	25-34 years	1,199 (60.0%)	994 (61.2%)	205 (54.7%)	
	≧35 years	288 (14.4%)	234 (14.4%)	54 (14.4%)	
Monthly household income[2]	x100,000VND	50.7 (34.7)	51.6 (35.9)	44.8 (26.1)	0.005
Mother's education level	More than 6 years	1,601 (80.1%)	1,316 (81.0%)	285 (81.0%)	0.028
Siblings	Yes	996 (49.8%)	841 (51.8%)	220 (58.7%)	<0.001
Paternal smoking	Yes	1,158 (57.9%)	930 (57.3%)	228 (60.8%)	0.211
Maternal smoking	Yes	0 (0%)	0 (0%)	0 (0%)	
Weight gain during pregnancy	<10 kg	553 (27.7%)	439 (27.0%)	114 (30.4%)	0.359
	10-15 kg	920 (46.0%)	758 (46.7%)	162 (43.2%)	
	≧15 kg	526 (26.3%)	427 (26.3%)	99 (26.4%)	
Mode of delivery[3]	NVD	747 (37.4%)	598 (36.8%)	149 (39.7%)	0.076
	CS	837 (41.9%)	699 (43.0%)	138 (36.8%)	
	Induction	415 (20.8%)	327 (20.1%)	88 (23.5%)	
Anemia during pregnancy (Hb level<11 mg/dl)[4]	Yes	521 (26.1%)	416 (25.6%)	105 (28.0%)	0.343
Maternal BMI[5]	<18.5 kg/m^2	530 (26.5%)	418 (25.7%)	112 (29.9%)	0.235

[1] p-values were tested using t-tests for the continuous variable (household income) and Chi-square tests for the categorical variables between followed-up and lost to follow-up groups.

[2] VND: Vietnamese dong

[3] NVD: Normal vaginal delivery, CS: Caesarean section.

[4] Hb: haemoglobin.

[5] BMI: body mass index.

Supplementary Table S2. Risk factors of all infections, lower respiratory tract infections and gastrointestinal infections in the Nha Trang Birth Cohort

	**All infections**				**Lower respiratory tract infections**				**Gastrointestinal infections**			
Variables	Unadjusted RR[1]	p-value	Adjusted RR[2]	p-value	Unadjusted RR	p-value	Adjusted RR[3]	p-value	Unadjusted RR	p-value	Adjusted RR[4]	p-value
Paternal smoking exposure during pregnancy and infancy												
Yes	**1.22 (1.02-1.46)**	**0.032**	**1.26 (1.05-1.51)**	**0.012**	**1.81 (1.30-2.52)**	**<0.001**	**1.76 (1.24-2.51)**	**0.002**	1.06 (0.80-1.41)	0.681	1.05 (0.79-1.39)	0.748
Sex												
Girls	0.89 (0.75-1.06)	0.199	0.91 (0.76-1.08)	0.268	0.82 (0.60-1.12)	0.221	0.82 (0.59-1.14)	0.244	0.82 (0.62-1.09)	0.164	0.82 (0.62-1.09)	0.166
Age at hospitalization												
0-5 months	**1 (reference)**		**1 (reference)**		1 (reference)		1 (reference)		**1 (reference)**		**1 (reference)**	
6-11 months	**2.25 (1.79-2.84)**	**<0.001**	**2.25 (1.79-2.83)**	**0.003**	1.25 (0.87-1.79)	0.615	1.10 (0.75-1.60)	0.910	**2.88 (1.79-4.62)**	**<0.001**	**2.98 (1.85-4.79)**	**<0.001**
12-17 months	**2.03 (1.61-2.57)**		**2.03 (1.61-2.57)**		1.11 (0.77-1.61)		1.04 (0.71-1.53)		**2.79 (1.73-4.49)**		**2.79 (1.73-4.49)**	
18-23 months	**1.62 (1.27-2.06)**		**1.61 (1.26-2.06)**		1.02 (0.70-1.49)		0.95 (0.64-1.41)		**1.87 (1.13-3.11)**		**1.93 (1.16-3.20)**	
Calendar month at hospitalization												
Jan-Mar	1 (reference)		**1 (reference)**		1 (reference)		1 (reference)		**1 (reference)**		**1 (reference)**	
Apr-Jun	1.13 (0.91-1.40)	0.065	**1.10 (0.89-1.37)**	**< 0.001**	1.11 (0.77-1.60)	0.660	0.97 (0.65-1.44)	0.475	**0.81 (0.52-1.26)**	**0.038**	**0.79 (0.51-1.22)**	**0.026**
Jul-Sep	1.32 (1.08-1.62)		**1.31 (1.06-1.61)**		1.20 (0.84-1.72)		1.24 (0.86-1.80)		**1.08 (0.73-1.62)**		**1.07 (0.72-1.60)**	
Oct-Dec	1.15 (0.93-1.42)		**1.16 (0.94-1.43)**		0.98 (0.67-1.43)		0.96 (0.64-1.42)		**1.44 (0.99-2.10)**		**1.45 (1.00-2.12)**	
Low birth weight												
Yes	1.38 (0.80-2.40)	0.253			2.14 (0.93-4.96)	0.080	2.27 (0.96-5.35)	0.062	1.43 (0.63-3.24)	0.414		
Monthly household Income												
per 100,000 VND	0.97 (0.85-1.11)	0.674			0.80 (0.62-1.02)	0.076	0.94 (0.88-1.00)	0.083	1.08 (0.87-1.35)	0.498		
Sibling												
Yes	**0.79 (0.67-0.95)**	**0.011**	0.87 (0.72-1.06)	0.165	0.95 (0.69-1.29)	0.734			0.87 (0.66-1.16)	0.344		
Maternal age at delivery												
<25 years	**1 (reference)**		1 (reference)		1 (reference)		1 (reference)		1 (reference)			
25-34 years	**0.79 (0.64-0.97)**	**0.010**	0.88 (0.71-1.09)	0.147	0.77 (0.54-1.09)	0.100	0.94 (0.64-1.37)	0.130	0.78 (0.57-1.07)	0.313		
≧35 years	**0.65 (0.48-0.87)**		0.72 (0.52-1.00)		0.57 (0.33-0.98)		0.57 (0.32-1.02)		0.80 (0.50-1.26)			
Anemia during pregnancy (Hb level<11g/dl)												
Yes	0.92 (0.75-1.13)	0.403			0.86 (0.59-1.24)	0.406			0.97 (0.70-1.34)	0.859		
Maternal BMI												
<18.5 kg/m^2	1 (reference)		1 (reference)		1 (reference)				1 (reference)			
18.5-23.5 kg/m^2	0.80 (0.66-0.98)	0.091	0.85 (0.70-1.04)	0.280	0.87 (0.61-1.24)	0.654			0.81 (0.59-1.11)	0.162		
≧23.5 kg/m^2	0.84 (0.58-1.20)		0.92 (0.64-1.33)		1.06 (0.57-1.96)				1.21 (0.73-2.02)			
Weight gain during pregnancy												
<10kg	1 (reference)				1 (reference)				1 (reference)			
10-15kg	0.94 (0.76-1.16)	0.821			0.91 (0.62-1.32)	0.876			0.95 (0.68-1.32)	0.351		
≧15kg	0.95 (0.75-1.21)				0.96 (0.63-1.47)				0.76 (0.51-1.13)			
Mode of delivery [4]												
NVD	1 (reference)				1 (reference)				1 (reference)			
CS	0.98 (0.80-1.20)	0.677			0.99 (0.70-1.42)	0.769			1.03 (0.75-1.42)	0.975		
Induction	1.09 (0.86-1.38)				1.15 (0.75-1.76)				1.00 (0.68-1.49)			
Mother's education level												
Low education (< 6 years)	1.38 (0.80-2.40)	0.253			1.36 (0.94-1.98)	0.107			1.43 (0.63-3.24)	0.410		

Statistically significant results are shown in **bold**; 95% confidence intervals of point estimates are in parentheses.

[1] Unadjusted rate ratio

[2] Rate ratios mutually adjusted for paternal smoking exposure during pregnancy and infancy, sex, age at hospital admission, calendar month, living with sibling, maternal age at delivery and maternal BMI.

[3] Rate ratios mutually adjusted for paternal smoking exposure during pregnancy and infancy, sex, age at hospital admission, calendar month, low birth weight, monthly household income and maternal age at delivery.

[4] Rate ratios mutually adjusted for paternal smoking exposure during pregnancy and infancy, sex, age at hospital admission, and calendar month.

Supplementary Table S3. Risk factor analysis of non-focal viral infections, upper respiratory tract infections and non-infectious diseases

	Non-focal viral infections				Upper respiratory tract infections				Non-infectious diseases			
Variables	Unadjusted RR[1]	p-value	Adjusted RR[2]	p-value	Unadjusted RR[1]	p-value	Adjusted RR[3]	p-value	Unadjusted RR[1]	p-value	Adjusted RR[3]	p-value
Paternal smoking exposure during pregnancy and infancy												
Yes	0.83 (0.60-1.16)	0.283	0.87 (0.63-1.22)	0.424	1.15 (0.79-1.67)	0.458	1.18 (0.81-1.72)	0.377	1.25 (0.90-1.74)	0.198	1.24 (0.89-1.72)	0.206
Sex												
Girls	1.17 (0.84-1.62)	0.362	1.15 (0.83-1.60)	0.400	0.74 (0.51-1.07)	0.109	0.75 (0.52-1.08)	0.126	0.70 (0.50-0.97)	0.100	0.74 (0.53-1.02)	0.063
Age at hospitalization												
0-5 months	**1 (reference)**		**1 (reference)**		**1 (reference)**		**1 (reference)**		1 (reference)		1 (reference)	
6-11 months	**3.25 (1.89-5.6)**	**<0.001**	**3.15 (1.83-5.44)**	**<0.001**	**3.16 (1.69-5.9)**	**<0.001**	**3.12 (1.67-5.82)**	**0.001**	0.76 (0.51-1.14)	0.496	0.76 (0.51-1.15)	0.519
12-17 months	**2.78 (1.59-4.83)**		**2.78 (1.59-4.84)**		**3.32 (1.79-6.17)**		**3.32 (1.79-6.17)**		0.98 (0.67-1.43)		0.98 (0.67-1.44)	
18-23 months	**2.6 (1.48-4.55)**		**2.54 (1.45-4.44)**		**2.31 (1.21-4.43)**		**2.28 (1.19-4.38)**		0.85 (0.58-1.26)		0.86 (0.58-1.27)	
Calendar month at hospitalization												
Jan-Mar	**1 (reference)**		1 (reference)		1 (reference)		1 (reference)		1 (reference)		1 (reference)	
Apr-Jun	**1.62 (1-2.63)**	**0.005**	1.57 (0.97-2.55)	**0.007**	1.17 (0.72-1.88)	0.753	1.14 (0.71-1.84)	0.815	1.01 (0.67-1.54)	0.712	1.03 (0.68-1.56)	0.739
Jul-Sep	**2.2 (1.39-3.47)**		2.15 (1.36-3.39)		0.99 (0.6-1.62)		0.97 (0.59-1.59)		1.18 (0.79-1.76)		1.19 (0.80-1.77)	
Oct-Dec	**1.36 (0.83-2.24)**		1.38 (0.83-2.27)		0.89 (0.54-1.48)		0.9 (0.54-1.49)		1.20 (0.81-1.79)		1.19 (0.80-1.77)	
Low birth weight												
Yes	0.56 (0.13-2.4)	0.399			1.1 (0.33-3.66)	0.884			1.46 (0.56-3.85)	0.716		
Monthly household Income												
per 100,000 VND	0.98 (0.91-1.06)	0.607			0.98 (0.91-1.06)	0.607			0.87 (0.68-1.12)	0.290		
Sibling												
Yes	**0.65 (0.46-0.9)**	**0.010**	**0.65 (0.45-0.94)**	**0.024**	**0.60 (0.42-0.87)**	**0.007**	0.73 (0.49-1.11)	0.138	0.75 (0.54-1.04)	0.073	0.87 (0.61-1.25)	0.452
Maternal age at delivery												
<25 years	1 (reference)		1 (reference)		**1 (reference)**		**1 (reference)**		1 (reference)		1 (reference)	
25-34 years	0.91 (0.62-1.33)	0.838	1.12 (0.74-1.68)	0.665	**0.65 (0.44-0.96)**	**0.002**	**0.75 (0.49-1.15)**	**0.037**	0.73 (0.51-1.04)	0.035	0.78 (0.53-1.14)	0.060
≧35 years	0.86 (0.50-1.50)		1.33 (0.72-2.44)		**0.30 (0.14-0.65)**		**0.37 (0.16-0.84)**		0.43 (0.24-0.79)		0.47 (0.24-0.89)	
Anemia during pregnancy (Hb level<11g/dl)												
Yes	0.8 (0.53-1.18)	0.253			1.11 (0.74-1.67)	0.624			0.77 (0.52-1.13)	0.222		
Maternal BMI												
<18.5 kg/m^2	**1 (reference)**		1 (reference)		1 (reference)				1 (reference)			
18.5-23.5 kg/m^2	**0.74 (0.52-1.04)**	**0.037**	0.77 (0.54-1.1)	0.084	0.7 (0.48-1.03)	0.132			1.15 (0.78-1.68)	0.830		
≧23.5 kg/m^2	**0.20 (0.03-1.48)**		0.22 (0.03-1.65)		0.51 (0.12-2.15)				1.07 (0.54-2.12)			
Weight gain during pregnancy												
<10kg	1 (reference)				1 (reference)				1 (reference)			
10-15kg	1.03 (0.68-1.55)	0.328			0.94 (0.61-1.47)	0.923			0.93 (0.64-1.36)	0.248		
≧15kg	1.34 (0.86-2.08)				1.03 (0.63-1.68)				0.77 (0.50-1.21)			
Mode of delivery [4]												
NVD	1 (reference)				1 (reference)				1 (reference)			
CS	0.95 (0.66-1.38)	0.945			1.00 (0.66-1.51)	0.954			1.07 (0.74-1.56)	0.193		
Induction	1.02 (0.65-1.6)				1.07 (0.65-1.77)				1.54 (1.01-2.35)			
Mother's education level												
Low education (< 6 years)	0.88 (0.57-1.36)	0.569			0.84 (0.52-1.38)	0.490			1.00 (0.67-1.51)	0.982		

Statistically significant results are shown in **bold**. 95% confidence intervals of point estimates are in parentheses.

[1]Unadjusted rate ratio

[2] Rate ratios mutually adjusted for paternal smoking exposure during pregnancy and infancy, sex, age at hospital admission, calendar month, living with sibling, maternal age at delivery and maternal BMI..

[3] Rate ratios mutually adjusted for paternal smoking exposure during pregnancy and infancy, sex, age at hospital admission, calendar month, living with sibling and maternal age at delivery.

Supplementary Table S4. Smoking status of adult male (20-44 year old in 2006) dwelling in Nha Trang (N=43,144), Vietnam

smoking in adult male (20-44yo) in 2006[1]	smoking in adult male (24-48yo) in 2010		
	yes	no	total
yes	22.4%	24.3%	46.7%
no	12.5%	40.8%	53.3%
total	34.9%	65.1%	100.0%

A cross-sectional surveillance of 74,228 households in 33 urban and rural communities (communes) in Khanh Hoa Province to collect information about the demographics, socioeconomic status and smoking habits of household members; the data were collected from the heads of households by trained interviewers during June and July of 2006. Census data was updated by interview in 2010 (See “Methods”). Patterns of change in smoking status of 43,144 adult male aged 20-44 years at first surveillance are tabulated. Note that changes in smoking status are rather less frequent, although the smokers decreased in number in this four years interval.
